# Colour Constancy for Image of Non-Uniformly Lit Scenes †

**DOI:** 10.3390/s19102242

**Published:** 2019-05-15

**Authors:** Md Akmol Hussain, Akbar Sheikh-Akbari, Iosif Mporas

**Affiliations:** 1 School of Computing, Creative Technology and Engineering, Leeds Beckett University, Leeds LS1 3HE, UK; m.hussein7766@student.leedsbeckett.ac.uk; 2 Communications and Intelligent Systems Group, School of Engineering and Computer Science, University of Hertfordshire, Hatfield AL10 9EU, UK; i.mporas@herts.ac.uk

**Keywords:** charge-coupled device sensor, colour constancy, multi-illuminants, k-means segmentation, fusion

## Abstract

Digital camera sensors are designed to record all incident light from a captured scene, but they are unable to distinguish between the colour of the light source and the true colour of objects. The resulting captured image exhibits a colour cast toward the colour of light source. This paper presents a colour constancy algorithm for images of scenes lit by non-uniform light sources. The proposed algorithm uses a histogram-based algorithm to determine the number of colour regions. It then applies the K-means^++^ algorithm on the input image, dividing the image into its segments. The proposed algorithm computes the Normalized Average Absolute Difference (NAAD) for each segment and uses it as a measure to determine if the segment has sufficient colour variations. The initial colour constancy adjustment factors for each segment with sufficient colour variation is calculated. The Colour Constancy Adjustment Weighting Factors (CCAWF) for each pixel of the image are determined by fusing the CCAWFs of the segments, weighted by their normalized Euclidian distance of the pixel from the center of the segments. Results show that the proposed method outperforms the statistical techniques and its images exhibit significantly higher subjective quality to those of the learning-based methods. In addition, the execution time of the proposed algorithm is comparable to statistical-based techniques and is much lower than those of the state-of-the-art learning-based methods.

## 1. Introduction

Image sensors built into today’s digital cameras mostly use either the Charge Coupled Device (CCD) or Complementary Metal-Oxide Semiconductor (CMOS) technology. Both CCD and CMOS are semiconductor devices that serve as “electronic eyes”. While they both use photodiodes, they differ in terms of manufacturing. A CCD sensor is an array of capacitors, each of which can store its own electrical charge. Groups of capacitors on the CCD form pixels, which are charged using the photoelectric effect. This happens when the capacitor converts an incident photon of light into an electrical charge. When a circuit is connected to the pixels, the value of the charge is then numerically measured and recorded in a computer file. After recording, the image can be displayed. Most CCD sensors use a Bayer filtration pattern for their pixels; each pixel is covered with either a red, blue, or green filter, which only allow light that is that colour to reach the capacitor. In this filtration pattern, there are twice as many green sensors as red or blue, because human eyes are more sensitive to green than to other colours. Since it is known which sensor has which colour filter, the intensity of red, green, and blue light can be determined anywhere on the CCD sensor [1]. The formed raw image using the red, green and blue signals generates the 16 million colours of the RGB colour space. The same principle is used in some other image sensors when they use a single-layer colour array [2].

CMOS sensors are much cheaper to produce than CCD sensors, as they are made of the same semiconductor fabrication lines used in microprocessor and static RAM memory chips. Consequently, they have largely replaced CCD sensors, which rely on more specialised fabrication methods. Each photosensitive pixel within the CMOS sensor is comprised of a photodiode and three transistors. One transistor is responsible for activating and resetting the pixel, the second amplifies and converts the stored charge within the photodiode to voltage and the third performs selection and multiplexing. The signals within each pixel are multiplexed by row and column to multiple on chip analog-to-digital converters, resulting in a high-speed yet low-sensitivity image capture system. Furthermore, CMOS sensors are susceptible to high fixed-pattern noise due to manufacturing flaws in the charge to voltage conversion circuits. As a result of their multiplexing design, CMOS sensors are often paired with electronic rolling shutters, although versions with additional transistors can be used in conjunction with global shutters and simultaneous exposure. The low sensitivity of a CMOS sensor results in a lower power consumption and the ability to handle higher light levels than a CCD sensor, resulting in their use in special high dynamic range cameras [3]. CCD and CMOS sensors are susceptible to different problems such as CCD sensors are more susceptible to vertical smear from bright light sources, while CMOS sensors are susceptible to skewing, wobbling and partial exposure. Research has shown that CCD and CMOS camera sensors are unable to recognise all colours within the scene [4].

Furthermore, digital camera sensors are designed to record all incident light from the scene but they are unable to distinguish between the colour of the light source and the true colour of objects. This results in the captured image exhibiting a colour cast, representing the colour of the light source [5,6,7,8,9]. Consequently, the colour constancy problem is underconstrained, and determining the true colour of objects when the scene is illuminated by non-canonical light sources is a challenge, so digital cameras use colour constancy adjustment techniques to estimate the true colour of objects [10,11,12]. The primary aim of all colour constancy adjustment algorithms is to remove the colour cast from digital images caused by the colour of scene illuminants [13,14,15] In this paper, existing colour constancy algorithms are discussed and a method to address colour correction in images containing large uniform colour areas and/or images of scenes illuminated by multiple light sources are presented, which produces significantly higher colour constancy than in the existing state-of-the-art methods.

### Related Work

Researchers have proposed various colour constancy adjustment methods to address the problem of colour constancy in the presence of both single and multiple light sources in digital images [16,17,18,19,20,21,22,23,24,25,26,27,28,29,30,31,32,33,34,35,36,37,38,39,40,41,42,43,44,45]. The existing colour constancy techniques can be grouped into five categories: statistics-based, gamut-based, physics-based, learning-based and biologically inspired methods. In this section, the key colour constancy adjustment methods are discussed.

The Grey World [16], the Max-RGB [17] and the Shades of Grey [18] are the main statistical-based colour constancy methods. These techniques are based on some assumptions on the statistics of the image data, such as achromaticity. Van de Weijer et al. [19] proposed the Grey Edge hypothesis, which assumes that the average edge difference of a scene’s image data is achromatic. The authors developed a framework that unified the aforementioned methods, which is shown in Equation (1):

(1) $${\left(\int \left|\frac{\partial^{n}f_{c,\sigma }\left(x\right)}{\partial x^{n}}\right|dx\right)}^{1/p}=ke_{c}^{n,p,\sigma }$$

where |·| indicates the Forbenius norm, *c* is the image colour component and c={R,G,B}, *n* is the order of the derivative, *p* is the Minkowski-norm, and $f_{c,\sigma }=f_{c}\times G_{\sigma }$ is the convolution of the image with a Gaussian filter with scale parameter σ.

Equation (1) can be used to represent different statistical colour constancy algorithms by using various values of *p*. When *p* = 1, Equation (1) becomes the Grey World method, which assumes the average value of all colour components within an image are achromatic. If *p* = ∞, Equation (1) represents the Max-RGB colour constancy algorithm, which assumes that the maximum values of the image colour components are achromatic. By setting *p* to be equal to 6, Equation (1) becomes the Shades of Gray algorithm, which is less data dependent than both the Gray World and White Patch colour correction methods. Equation (1) can also represent higher order colour constancy methods, including Grey Edge-1 and Grey Edge-2 by setting *p =* 1, σ=1 and *n* = 1 or 2, respectively. These two approaches assume that derivatives of the image colour components are achromatic. The Weighted Grey Edge method was proposed by Gisenji et al. in [20]. This colour correction technique is an extension of the Grey Edge algorithm, which incorporates the general weighting scheme of the Grey Edge method as well as the edge of the shadows within the image to achieve colour correction. A moment-based colour balancing method, which uses several higher order moments of the colour features on a fixed 3 × 3 matrix transformation, was also introduced by Finlayson in [21].

Forsyth [22] introduced the gamut mapping method, which assumes that only a limited number of colours can be observed for any given illuminant in real-world images. Gijsenij et al. [23] proposed an extended version of gamut mapping by incorporating the differential nature of the image. The authors have shown that the failure of the diagonal model of the gamut mapping framework can be barred by adjusting the diagonal-offset model proposed in [24].

Several learning-based colour constancy adjustment methods have recently been reported in the literature. The colour constancy problem as a 2D spatial localization task in a log-chrominance space was formulated by Baron in [25]. Baron observed that scaling the image colour components induces a translation in the log-chromaticity histogram of the image, allowing a colour constancy adjustment to be performed using learning-based methods such as Convolutional Neural Network (CNN). A light source estimation algorithm, which employs a CNN that contains one convolutional layer, one fully connected layer and three output nodes was proposed by Bianco et al. in [26]. The proposed CNN-based technique samples several non-overlapping patches from the input image and then applies a histogram-stretching algorithm to neutralize the contrast of the image. It then fuses the patch scores, which are obtained by extracting activation values of the last hidden layer to guess the light source. They reported satisfactory performance of the algorithm on a specific raw image dataset. An extension of this neural network-based colour correction method was introduced by Fourure et al. in [27], which uses a mix-pooling method to determine the availability of accurate features to be learned to perform colour correction. Another CNN-based method, which determines the number of image scene illuminants using a Kernel Density Estimator (KDE) algorithm, was proposed in [28]. The proposed technique assigns local estimations to the density peaks to compute supports for local regression and back-project the results to the original image based on a distance transform. Finally, the estimated illuminants are refined by non-linear local aggregation to produce a universal estimate for each scene illuminant. Qian et al. [29] proposed the recurrent colour constancy network (RCC-Net), which consists of two parallel convolutional long-term temporal memory networks, to process the original frame sequence and simulated spatial sequence to learn compositional representations in space and time. This end-to-end RCC-Net is equipped with a simulated sequence module, which boosts its performance for temporal colour constancy task.

To perform colour constancy for images of scenes illuminated by multiple light sources, researchers have proposed techniques which estimate the local incident light based on the assumption that the colour of the incident light of a mini region is uniform [30,31,32,33]. Riess et al. [30] have estimated the source light for each mini region of superpixels using a graph-based algorithm. Their approach creates an illuminant map that is coloured by the local estimates and merges areas with similar light colour together using the quick shift algorithm to obtain a new estimate for each region. In a comparable work, Blier et al. have applied five state-of-the-art algorithms to estimate the local illuminant for superpixels of areas of approximately similar colour [31]. They choose the best estimates based on the error statistics and combine the estimates for per superpixel using a machine-learning-based regression algorithm. Mazin et al. [34] proposed a technique to extract a set of gray pixels from the image to estimate a set of possible light source for the pixels by using the Planckian locus of black-body radiators. In [35], Bianco and Schettini proposed a method to estimate the colour of the light from human faces within the image by using a scale-space histogram filtering method. Their algorithm performs well; however, its application is limited to images that contain a clear visible human face. In [36], a 3D scene geometry model was created by applying hard and soft segmentation methods to the input image. It then links the statistical information of the different layers of the model to suitable colour constancy techniques. A colour balancing technique was proposed in [37] that employs a diagonal 3 × 3 matrix on the ground truth illuminant of a 24-colour patch, the method of which generates superior results compared to other diagonal algorithms. Arguing the robustness of the abovementioned methods, a user-guided colour correction method for multiple illuminants was proposed in [38].

A biologically inspired model for colour constancy was reported by Gao et al. [39] that exploits the response of the Double Opponent cells in different channels and estimates the colour of the illuminant by max or sum pooling mechanism in long, medium and short wavelength colour space. An improved retinal-mechanism-based model was proposed by Zhang et al. [40]. The authors imitated the function of Horizontal Cell (HC) modulation that provides global colour correction with cone-specific lateral gain control. Akbarnia and Parraga [41] proposed a colour constancy model using two overlapping asymmetric Gaussian kernels, where the contrast of the surrounding pixels is used to adjust the kernels’ sizes (approximating the change of visual neuron’s receptive field size). Finally, the outputs of the most activated visual neuron’s receptive fields are used to estimate the colour of the light.

Yang et al. [42] proposed a grey pixel-based colour constancy method by using the illuminant-invariant measure. Joze and Drew [43] showed that the texture feature of an image is ideal candidate for illuminant estimation. Their approach took the weakly colour constant RGB values from the texture to find the neighbour surface based on histogram matching from the training data. Male et al. [44] employed an automatic human eyes detection method and extracted the scelera pixels to estimate the scenes’ illuminant.

The aforementioned algorithms work reasonably well when the scene is illuminated by a uniform light source and lacks large uniform colour areas within the image, but the performance of existing algorithms deteriorates in the presence of large uniform colour areas and when the scene is illuminated by multiple non-uniform light sources. This paper presents a colour constancy algorithm for images of scenes lit by multiple non-uniform light sources (this paper is an extended version of a previous paper [45]). The proposed algorithm first applies a histogram-based approach to determine the number of segments that represent different colour variations of the image. The K-means^++^ clustering method [46] is then used to divide the input image into this number of segments. The proposed method then calculates the Normalized Average Absolute Difference (NAAD) of each resulting segment and uses it as a criterion to discard segments of near-uniform colour, which could potentially bias the colour-balanced image toward those colours. The initial colour constancy adjustment weighting factors for each of the remaining segments are computed based on the assumption that the achromatic values of the colour components of the segment are neutral. The colour constancy adjustment weighting factors for each pixel are finally determined by fusing the colour constancy of all selected segments adjusted by the normalized Euclidian distance of the pixel from the centroids of the selected segments. Experimental results on the images of five benchmark standard image datasets show the merit of the proposed algorithm over existing techniques. The rest of this paper is organized as follows: In Section 2, the proposed algorithm is described, experimental results and their evaluation are given in Section 3 and Section 4 concludes the paper.

## 2. Image Colour Constancy Adjustment by Fusion of Image Segments’ Initial Colour Correction Factors

The proposed Colour Constancy Adjustment by Fusion of Image Segments’ initial colour correction factors (CCAFIS) algorithm is divided into three steps: automatic image segmentation, segment selection and calculation of initial colour constancy weighting factors for each segment and calculation of the colour adjustment factors for each pixel. Each of these three steps is detailed in the following sub-sections.

### 2.1. Automatic Image Segmentation

The proposed automatic image segmentation algorithm first converts the input RGB image into a grey image. It then splits the coefficients within the resulting grey image into a histogram with 256 bins. The resulting histogram is then filtered using the following Gaussian low-pass filter: $\left[\begin{array}{ccc} c_{1} & c_{2} & \begin{array}{ccc} \ldots & c_{6} & \begin{array}{ccc} c_{7} & c_{6} & \begin{array}{cc} \ldots & c_{1} \end{array} \end{array} \end{array} \end{array}\right]$, where $c_{1}$ to $c_{7}$ are equal to 0.0002, 0.0029, 0.0161, 0.0537, 0.1208, 0.1934 and 0.2256, respectively. The algorithm then counts the number of the local maxima found in the smoothed histogram to determine the required number of the segments for the proposed colour constancy algorithm. To do this, the minimum distance between two local maxima and the minimum local maxima height were set to 0.05 and 0.001^th^ of the total number of image pixels, respectively. These numbers were empirically found to be effective in finding the reasonable number of segments when dealing with images of five different image datasets. The calculated number of segments and the ${La}^{*}b^{*}$ format of the input RGB image are then fed into a K-means^++^ clustering algorithm, which divides the input image pixels into several segments based on their colour properties.

### 2.2. Segments’ Selection and Calculation of Initial Colour Constancy Weighting Factors for each Segment

In this section, each of the resulting segments are processed independently to circumvent the segments containing uniform areas and to calculate initial colour adjustment factors for each segment, as follows:

The proposed technique first calculates the Normalized Average Absolute Difference (NAAD) of pixels for each colour component of the segment using Equation (2):

(2) $${\mathrm{NAAD}}_{C}=\{\begin{array}{c} \frac{1}{T\times {\bar{F}}_{C}}\sum \left(\left|\begin{array}{ccc} F_{C}\left(x,y\right) & - & {\bar{F}}_{C} \end{array}\right|\right)\\ x,\;y\in segment \end{array}$$

where $NAAD_{C}$ is the Normalized Average Absolute Difference of the segments colour component C, C∈{R,G,B}, T is the total number of pixels in the segment, $F_{C}\left(x,y\right)$ represents component C’s coefficients of the segment at location x and y and ${\bar{F}}_{C}$ is the average value of the component C of the segment’s coefficients.

The resulting $NAAD_{C}$ value is compared with an empirically pre-determined threshold value for that colour component. The threshold values for the colour components R, G and B are named $T_{R}$, $T_{G}$ and $T_{B}$, respectively. If the calculated NAAD values of the three colour components of the segment are greater than their respective threshold values, the segment represents a non-uniform colour area. Hence, this segment is selected to contribute to the colour correction of the whole image and a bit representing this segment within the Decision Vector (DV) is set. The proposed technique then calculates the initial colour adjustment factors for the selected segment using the Grey World algorithm [16], as written in Equation (3):

(3) kC=SmeanSCmean

where $k_{C}$ is component C’s initial colour adjustment factor, $S_{mean}$ is the average value of the segment’s coefficients, and $S_{C_{mean}}$ represents the average value of the segment’s component C’s coefficients, where C∈{R,G,B}.

In this research, the Grey World colour constancy method, which is one of the most effective and yet computationally inexpensive techniques compared to other statistical colour constancy algorithms [2,3], is used to compute the initial colour constancy weighting factors for segments. However, other statistical colour constancy methods can be used.

### 2.3. Calculation of the Colour Adjustment Factors for each Pixel

In this section, the fusion of the initially calculated colour constancy adjustment factors for the selected segments to calculate per pixel colour correction weighting factors is discussed. The algorithm is then fed the calculated initial colour constancy adjustment factors of the selected segments, the gravitational centroid of each selected segment’s pixels and the Decision Vector (DV). The proposed algorithm calculates the Euclidian distance of each pixel from the centers of the selected segments and uses them to regulate initially calculated colour adjustment factors to determine per pixel weighting factors using Equation (4):

(4) $$k_{Ci}=\frac{d_{1}}{d_{1}+d_{2}+\cdots +d_{n}}\;\left(k_{C1}\right)+\frac{d_{2}}{d_{1}+d_{2}+\cdots +d_{n}}\;\left(k_{C2}\right)+\cdots \;\;+\frac{d_{n}}{d_{1}+d_{2}+\cdots +d_{n}}\;\left(k_{Cn}\right)$$

where $k_{Ci}$ is the colour constancy adjustment factor for component C of the pixel i, C ∈{R,G,B}, *d*_1_, *d*_2_, …, and *d*_n_ are the Euclidian distance of the pixel i from the centroid of the segments 1, 2,.., *n*, respectively.

This balances the effect of colours of different light sources on the colour of each pixel. The resulting weighting factors are used to colour balance the input image using The Von-Kries Diagonal model [47], as shown in Equation (5):

(5) $$I_{out\_i}=\left(\begin{array}{ccc} K_{Ri} & 0 & 0\\ 0 & K_{Gi} & 0\\ 0 & 0 & K_{Bi} \end{array}\right)\left(\begin{array}{c} I_{Ri}\\ I_{Gi}\\ I_{Bi} \end{array}\right)$$

where $I_{out\_i}$ is the colour balanced pixel i, $K_{Ri}$, $K_{Gi}$, $K_{Bi}$ are the calculated weighting factors for pixel i and $I_{Ri}$, $I_{Gi}$, $I_{Bi}$ are the R, G and B colour components of the pixel i of the input image.

## 3. Experimental Results and Evaluation

In this section, the performance of the proposed Colour Constancy Adjustment by Fusion of Image Segments’ initial colour correction factors (CCAFIS) is assessed on five benchmark image datasets, namely: Multiple Light Source (MLS) [32], The Multiple Illuminant and Multiple Object (MIMO) [33], The Colour Checker [48], Grey Ball [49] and the UPenn Natural image dataset [50]. The Evaluation procedures are discussed in Section 3.1, an example of image segmentation and segment selection is given in Section 3.2 and the experimental results are discussed in Section 3.3.

### 3.1. Evaluation Procedure

The performance of the colour constancy algorithms are generally assessed both objectively and subjectively. The angular error, also known as the recovery angular error, is an objective criterion that is widely used to assess the colour constancy of the images which measures the distance between the colour-corrected image and its ground truth [51]. A lower resulting mean or median angular error of the images of an algorithm indicates that the algorithm’s performance is superior. The recovery angular error of an image can be calculated using Equation (6):

(6) $$d_{\theta \left(recovery\right)}=\cos^{-1}\left(\frac{e.\hat{e}}{\left\|e\|\|\hat{e}\right\|}\right)$$

where $e.\hat{e}$ shows the dot product of the ground-truth and the colour-corrected image vectors, respectively, and ‖.‖ is the Euclidian norm of the vector.

Recently, Finlayson et al. [52] have critiqued the application of the (recovery) angular error measure based on the argument that it produces different results for identical scenes viewed under different colour light sources. They proposed an improved version of the recovery angular error measure, called the reproduction angular error, which is defined as the angle between the image RGB of a white surface when the actual and estimated illuminations are ‘divided out’. The reproduction angular error metric can be calculated using Equation (7):

(7) $$d_{\theta \left(reproduction\right)}=\cos^{-1}\left(\frac{\left(\left\|e/\hat{e}\right\|\right)}{e/\hat{e}}.w\right)$$

where $w=\;\frac{e/\hat{e}}{\surd 3}$ is the true colour of the white reference.

Both recovery and reproduction angular error have been used to assess the objective quality of the colour-corrected image by computing the average of the mean or median recovery/reproduction angular errors of different methods on a large set of colour-corrected images and using them for comparison purpose. The images of the method that have the lowest average of the mean or median recovery/reproduction angular errors have the highest colour constancy.

Because human eyes are the final judge for assessing the colour constancy of images, a subjective evaluation is generally considered to be the most reliable assessment method when evaluating colour constancy algorithms. Mean Opinion Score (MOS) is a popular subjective evaluation method that is widely used to compare the visual quality of the images. To determine the MOS for images subjected to different colour constancy adjustment methods, a set of images of different scenes containing diverse backgrounds, foregrounds, objects and a range of colour variations that are taken under various lighting conditions is first chosen. These images are then colour-corrected using different colour balancing techniques. The resulting images are shown to observers who score the images based on their colour constancy (the same laptop was used to present the images to all observers). The MOS of each method finally calculated by computing the average score of its images.

Objective criteria are widely used to assess the performance of different colour constancy techniques due to their simplicity. However, there is significant debate on the merit of objective measurements and their relation to subjective assessment. Some researchers have argued that objective measurements may not always be in agreement with the subjective quality of the image [53,54]. Consequently, in this paper, both subjective and objective assessment methods have been used to assess the performance of the colour constancy algorithms.

### 3.2. Example of Image Segmentation and Segment Selection

**Figure 1:**
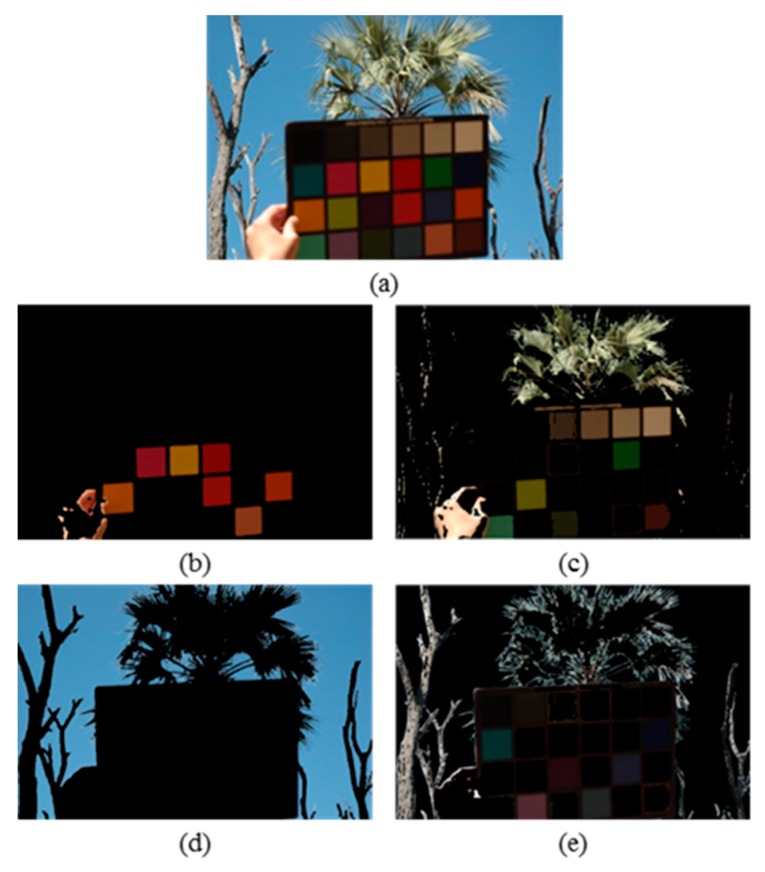
A sample image from the UPenn dataset [49] and its four resulting segments: (**a**) original image, (**b**–**e**) segment 1-4.

To visualize the effectiveness of the proposed method in dividing the image into several segments and identifying segments with uniform colour areas, a sample image from the UPenn images dataset [50] is taken and processed by the proposed method. Figure 1a shows the input image and Figure 1b–e show its resulting four segments, with the pixels excluded from each segment coloured black. From Figure 1, it is obvious that Figure 1d represents the segment with uniform colour areas.

The Normalized Average Absolute Differences (NAAD) of the three colour components of the segments of the image shown in Figure 1 have been calculated using Equation (2) and tabulated in Table 1. From this table, it is clear that the calculated NAAD values for the three colour components of segment 3 (highlighted with a green border), which represents the uniform colour area of the image, are below the empirically determined thresholds’ values. As a pre-processing step, extensive experiments were performed using the benchmark image dataset to empirically determine the threshold values. The results showed that a threshold value of 0.01 for the T_R_, T_G_ and T_B_ components can efficiently eliminate segments with uniform areas [55]. Hence, the proposed technique will exclude this segment’s coefficients from contributing into the colour correction of the whole image.

### 3.3. Experimental Results

In the next two sub-sections, subjective and objective results for the proposed method will be presented.

#### 3.3.1. Subjective Result

**Figure 2:**
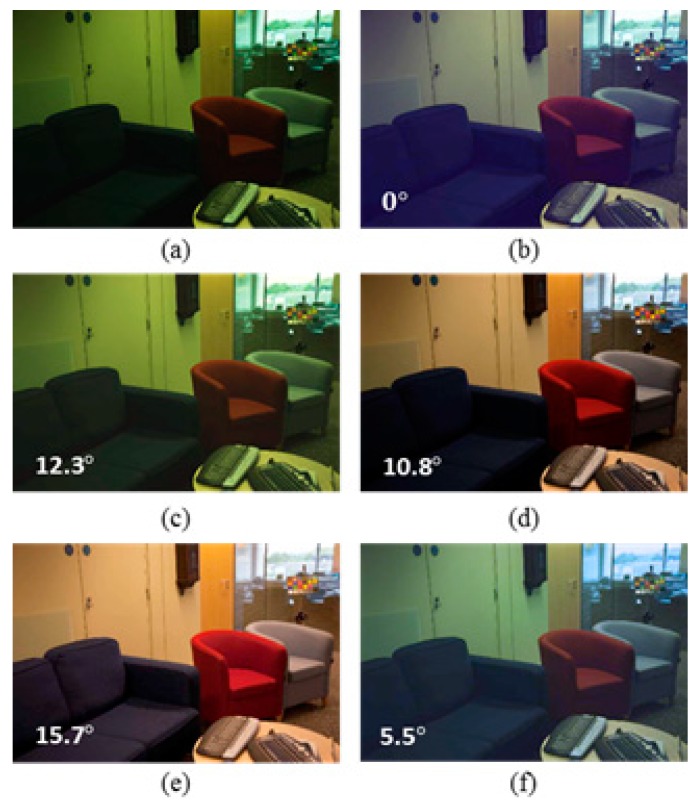
Original, ground truth and its colour-balanced images using different colour correction methods: (**a**) Original image from the Colour Checker dataset [48], (**b**) Ground truth image, (**c**) Weighted Grey Edge, (**d**) Corrected Moment, (**e**) Cheng et al. and (**f**) Proposed CCAFIS’ methods’ images.

To demonstrate the subjective performance of the proposed Colour Constancy Adjustment by Fusion of Image Segments’ initial colour correction factors (CCAFIS) method and to compare the quality of its colour-corrected images with those of the state of the art colour constancy techniques, two sample images from the Colour Checker and the Upenn Natural Image benchmark image datasets are selected and colour-balanced using different colour correction methods. Figure 2 shows a sample image from the Colour Checker image dataset, its corresponding ground truth and its colour-balanced images using the Weighted Grey Edge [20], Corrected Moment [21], Cheng et al. [38] and the proposed CCAFIS techniques. The resulting images have been linearly gamma-corrected to improve their visual qualities. From Figure 2a, it can be seen that the input image has a significant green colour cast and the scene is illuminated by multiple indoor and outdoor light sources. Figure 2b shows the ground truth of the image. Figure 2c shows the Weighted Grey Edge method’s image, which demonstrates a slightly lower green colour cast than the input image. Figure 2d illustrates the Corrected Moment’s image, which exhibits a yellow to orange colour cast. Cheng et al.’s method’s image is shown in Figure 2e. This image suffers from the presence of a deep yellow-orange colour cast. The proposed CCAFIS method’s image, shown in Figure 2f, exhibits high colour constancy and appears to have the closest colour constancy to that of the ground truth image. The recovery angular error of the images are also shown on the images; from these figures it can be seen that the recovery angular error of the proposed method’s image is the lowest among all other methods. This implies that the objective qualities of the images are consistent with their subjective qualities.

**Figure 3:**
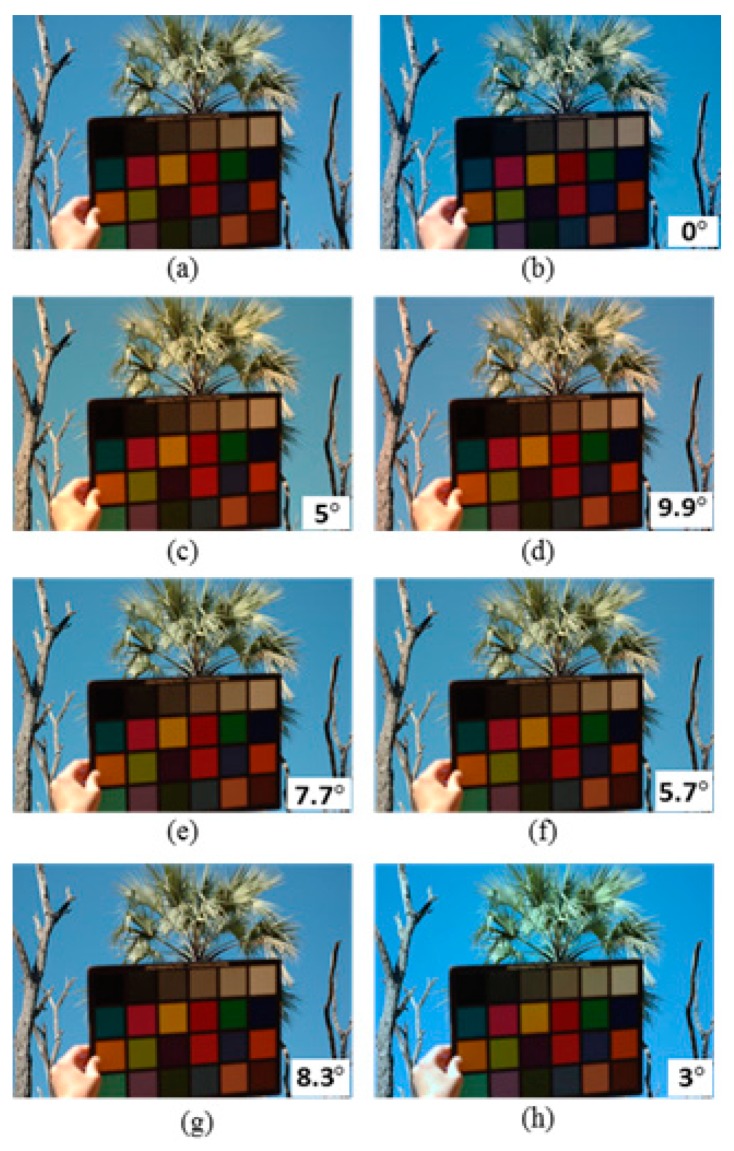
Original, ground truth and its colour-balanced images using different colour correction methods: (**a**) Original image from the UPenn Natural Image dataset [50], (**b**) Ground truth, (**c**) Max-RGB, (**d**) Shades of Grey, (**e**) Grey Edge-1, (**f**) Grey Edge-2, (**g**) Weighted Grey Edge and (**h**) Proposed CCAFIS’ methods’ images.

Figure 3 shows a sample image from the UPenn dataset [49], its ground truth and colour-balanced images using the Max-RGB, Shades of Grey, Grey Edge-1, Grey Edge-2, Weighted Grey Edge and the proposed CCAFIS’ methods’ images. From Figure 3a, it can be noted that the input image exhibits a yellow colour cast. The tree’s green leaves and the colour chart exhibit yellow colour cast. Figure 3b is its ground truth image. Figure 3c shows the Max-RGB method’s image. From this image, it can be seen that the image has a slightly higher yellow colour cast than its original input image. The Shades of Grey method’s image is shown in Figure 3d. This figure demonstrates significantly higher yellow colour cast than the original image, particularly on the tree’s green leaves area of the image. Figure 3e is the Grey Edge-1 method’s image. This image also suffers from an increased colour cast on the tree’s green leaves and the colour chart areas of the image. The Grey Edge-method’s image is shown in Figure 3f. This image demonstrates a slightly higher colour constancy than its original image. The tree and the deciduous plants on the left side of the image have a slightly lower colour cast than the original image. The Weighted Grey Edge method’s image is illustrated in Figure 3g. This image is appeared to be very alike to that of the Grey Edge-1 method’s image, shown in Figure 3e.

**Figure 4:**
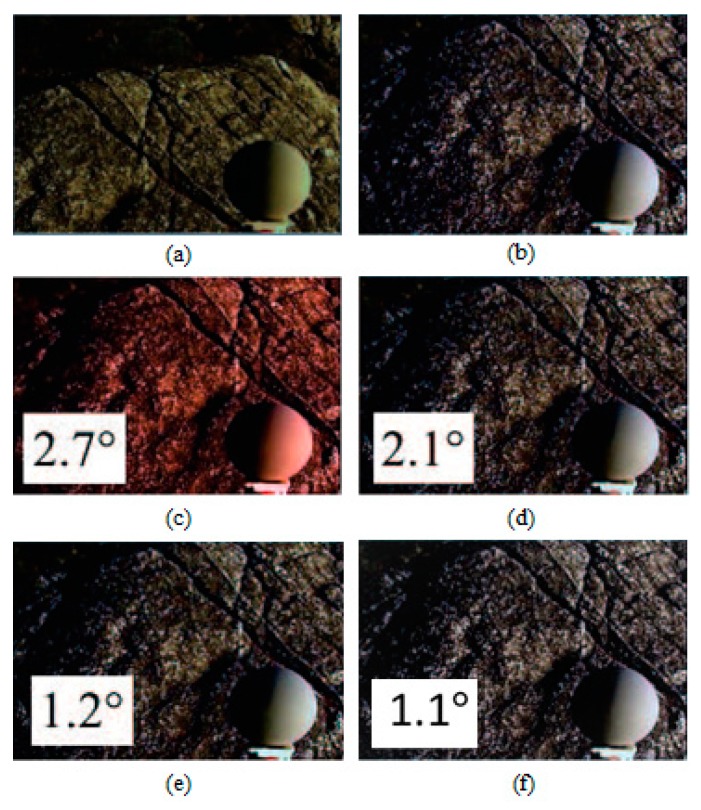
Original, ground truth and its colour-balanced images using different colour correction methods: (**a**) Original image from the Grey Ball dataset [49], (**b**) Ground truth, (**c**) Edge-based-gamut, (**d**) Grey Pixel, (**e**) RCC-Net, (**f**) Proposed CCAFIS’ methods’ images.

Figure 4 illustrates a sample image from the Gray Ball dataset with a yellow colour cast, its respective ground truth image and its colour-balanced images using Edge-based gamut [23], Grey pixel [42], RCC-Net [29], and the proposed CCAFIS methods’ images. From Figure 4c, it can be seen that the Gamut-based method exhibits a high level of red colour casts. Figure 4d and Figure 4e are the images of the Grey Pixel and the RCC-Net methods, respectively. These images demonstrate an improved colour balance to that of the input image. However, the images have still some levels of yellow colour cast. The proposed CCAFIS method’s image (Figure 4f) appears to be a shot under canonical light as the presence of the source illuminant is significantly reduced. Moreover, the median recovery angular error of the proposed technique’s image is the lowest among all other techniques’ images, which means the proposed technique’s image has the highest objective colour constancy.

**Figure 5:**
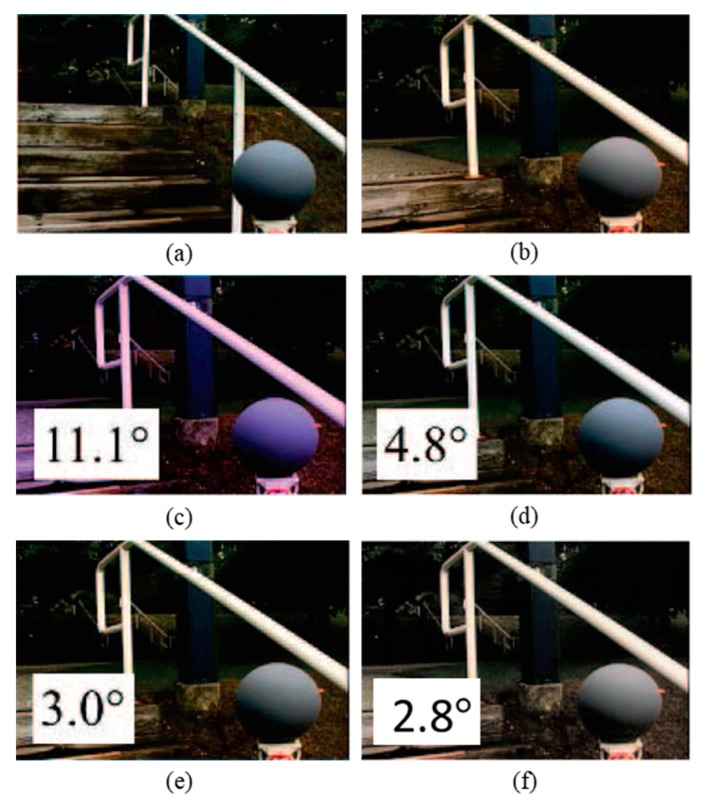
Original, ground truth and its colour-balanced images using different colour correction methods: (**a**) Original image from the Grey Ball dataset [49], (**b**) Ground truth, (**c**) Edge-based-gamut, (**d**) Grey Pixel, (**e**) RCC-Net, (**f**) Proposed CCAFIS’ methods’ images.

Figure 5 illustrates a sample image from the Gray Ball image dataset [49], which has a yellow colour cast, and its colour-balanced images using the Edge-based gamut [23], Grey pixel [42], RCC-Net [29] and the proposed CCAFIS methods’ images. From Figure 5c, it can be seen that the Gamut-based method’s image exhibits both blue and reddish colour cast. However, this image exhibits lower colour constancy to those of the Grey pixel and the RCC-Net techniques’ images, shown in Figure 5d and Figure 5e, respectively. Nevertheless, the images of all these three techniques still have visible yellow colour cast. The proposed CCAFIS method’s image (Figure 5f) appears as if being taken under a white illuminant. The recovery angular errors of the images are also calculated and displayed on the images. By comparing the images’ recovery angular error, it can be seen that the proposed method’s image has the lowest recovery angular error, which means it exhibits the highest objective colour quality to other techniques images.

**Figure 6:**
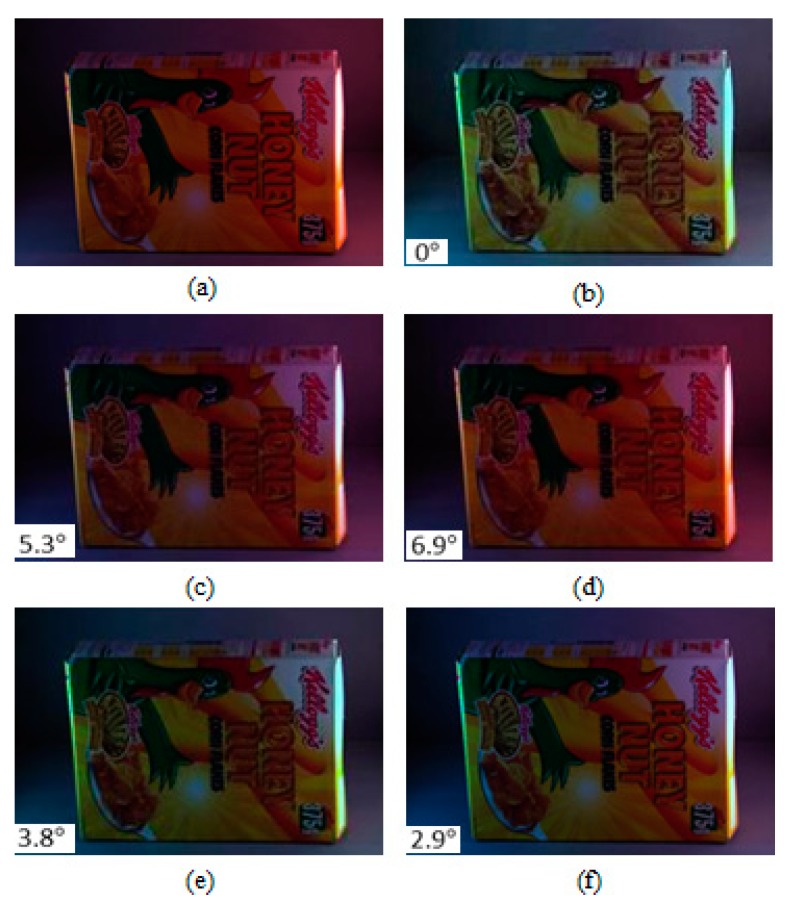
Original, ground truth and its colour-balanced images using different colour correction methods: (**a**) Original image from the MLS dataset [32], (**b**) Ground truth image, (**c**) Grey Edge-2, (**d**) Weighted Grey Edge, (**e**) Gisenji et al. and (**f**) Proposed CCAFIS methods’ images.

To give the reader a better understanding in the performance of the proposed CCAFIS method on images of scenes with spatially varying illuminant distribution, an image from the MLS image dataset [32] that represents a scene lit by spatially varying illumination is taken and colour corrected using the proposed technique, Grey Edge-2, Weighted Grey Edge and Gisenji et al. The original image, its ground truth and the resulting colour-corrected images using the proposed CCAFIS and other techniques are shown in Figure 6. From this figure, it can be noted that the proposed technique’s image exhibits the highest colour constancy. In addition, it has the lowest median angular error among all other techniques’ images, which implies that the proposed technique’s image has the highest objective quality.

To generate the Mean Opinion Score (MOS) for the images of the proposed and the state-of-the-art colour constancy methods, a set of images from the Grey Ball, the Colour Checker and the MIMO image datasets, which contain images of scenes lit by either single or multiple light sources, was chosen. The selected images were colour-corrected using the proposed CCAFIS method, as well as other state-of-the art techniques including the multiple illuminant methods such as: Gijsenij et al. [32], MIRF [33] and Cheng et al. [38]. Ten independent observers subjectively evaluated the resulting colour-balanced images. The viewers scored the colour constancy of each image from 1 to 5, where higher numbers correspond to increased colour constancy. The average MOS of different methods’ images were then calculated and tabulated in Table 2. From Table 2, it can be noted that the proposed method’s images have the highest average MOS when compared to the other techniques’ images. This implies that the proposed method’s images have the uppermost subjective colour constancy.

#### 3.3.2. Objective Result

To evaluate the objective performance of the proposed method, Grey world [16], Max-RGB [17], Grey Edge-1 [19], Grey Edge-2 [19], Gijsenij et al. [32], MIRF [33], ASM [41], Grey Pixel [42], Exemplar [43] and CNN+SVR [28] methods were used to colour balance the images of the MIMO [33], the Grey Ball [49] and the Colour Checker [48] image datasets, as well as 9 outdoor images of the Multiple light sources image dataset [32]. The average mean and median of both recovery and reproduction angular errors of the colour-balanced images of the Grey Ball and the Colour Checker dataset are tabulated in Table 3 and Table 4, respectively.

From Table 3, the proposed technique’s images have the lowest average mean and median recovery and reproduction angular errors among all the statistics-based colour constancy methods, implying that the proposed technique outperforms statistics- and gamut-based techniques with respect to objective colour constancy (the mean and the median angular errors of the Deep Learning, Natural Image Statistics and Spectral Statistics were taken from [41]). When compared to the learning-based methods, the proposed technique’s average median angular error equals 4.0°, which is slightly higher than that of the learning-based methods. The proposed algorithm’s median reproduction angular error is 2.6°, which is the lowest among all techniques apart from ASM with a median angular error of 2.3°. This demonstrates that the proposed method produces very competitive objective results compared to those of the learning-based methods.

According to Table 4, the proposed CCAFIS technique’s images have the lowest average mean and median recovery and reproduction angular errors among all the statistics-based colour constancy methods, which implies that the proposed technique outperforms statistics- and gamut-based techniques with respect to objective colour constancy. With respect to the learning-based methods, the proposed technique’s average median angular error equals 2.7°, which is slightly higher than some of the learning-based methods (the mean and the median angular error of the AAC, HLVIBU, HLVI BU & TD, CCDL, EB, BDP, CM, FB+GM, PCL, SF, CCP, CCC, AlexNet + SVR, CNN-Per patch, CNN average-pooling, CNN median-pooling and CNN fine-tuned methods have been taken from [28]). The proposed algorithm’s median reproduction angular error is 2.9°, which is the lowest among all techniques except the Exemplar-based method with a median angular error of 2.6°. This demonstrates that the proposed method produces very competitive objective results compared to those of the learning-based methods.

The average mean and median recovery angular errors of the colour-balanced images from the MIMO image dataset for different techniques were computed and tabulated in Table 5 and the median angular errors for 9 outdoor image of multiple light source dataset for different algorithms were determined and tabulated in Table 6.

From Table 5, it can be seen that the proposed CCAFIS method’s mean recovery angular error for real world images of the MIMO dataset is 4.2°. Please note that the mean and the median recovery angular error for the MLS + GW, MLS + WP, MIRF + GW, MIRF + WP and MIsRF + IEbV methods have been taken from [28]. This is the same as the Grey World’s and MLS + GW mean recovery angular error and slightly higher than that of the MIRF methods, which produced images having the smallest mean angular error of 4.1°. The median recovery angular error of the proposed CCAFIS method is 4.3°, which is the lowest among all of the statistics-based methods. For the laboratory images, the proposed CCAFIS method’s mean recovery angular error is 2.1° and the median recovery angular is 2.7°, which are the lowest recovery angular errors compared to all other methods. This implies that the proposed CCAFIS method has the highest objective performance when dealing with lab images of MIMO dataset.

From Table 6, it can be noted that the proposed CCAFIS method’s images exhibit the lowest median recovery angular error among all statistical and the state of art techniques. This implies that the proposed CCAFIS method outperforms other methods in adjusting the colour constancy of the images taken from scenes illuminated by multiple light sources.

## 4. Execution Time

To enable the reader to compare the execution time of the proposed Colour Constancy Adjustment by Fusion of Image Segments’ initial colour correction factors (CCAFIS) algorithm with both statistical-based and learning-based state of the art colour constancy techniques, the proposed method, Gray World, Max-RGB (White Patch), Gray Edge-1, Gray Edge-2, Exemplar-based [43], Gray Pixel (std) [42] and ASM [41] algorithms were implemented in MATLAB. They were then run on the same Microsoft Windows 10 based personal computer, running on Intel^®^ Core (TM) i3-6006U CPU with a 1.99 GHz processor, 4.00 GB of RAM, and without any additional dedicated graphic processing unit. These methods were timed when applied to colour balance the first 100 images of the Colour Checker benchmark image dataset. For the learning-based techniques, the second 100 images from the Colour Checker benchmark image dataset were used for training. The cumulative execution time for testing the proposed CCAFIS, statistical-based and learning-based methods were measured and tabulated in Table 7. From Table 7, it can be seen that the proposed CCAFIS technique requires slightly more execution time to colour balance the images in comparison to other statistical-based methods. This is consistent with the fact that the proposed technique calculates colour adjustment factors for each pixel of the input image separately. Moreover, this is the price for achieving higher colour constancy in the presence of large uniform colour patches within the image and when the scene is illuminated by multiple non-uniform light sources. However, the proposed method demonstrates very competitive colour correction at a significantly lower computation time when compared to the learning-based state of the art techniques. In summary, it can be concluded that the proposed CCAFIS algorithm provides significantly higher performance to those of statistical-based colour constancy adjustment techniques at slightly higher computational cost, while generating very competitive results that are comparable to those of the state-of-the-art learning-based methods, but at a hugely lower computational cost. In addition, the proposed CCAFIS technique is data independent and produces accurate results without prior knowledge of the image dataset, unlike the learning-based algorithms, which achieve high performance when dealing with the images of the dataset that was used for training. The performance of the learning-based techniques usually deteriorates when used for cross-dataset image testing.

## 5. Conclusions

This paper presented a colour constancy algorithm for non-uniformly lit scenes’ images. The algorithm uses the K-means^++^ clustering algorithm along with a histogram-based method to divide the input image into several segments with similar colour variations. The Normalized Average Absolute Difference (NAAD) of the resulting segments are then calculated and used as a measure to identify segments with uniform colour areas. These segments are then excluded from the calculation of colour constancy adjustment factors for the whole image. The initial colour constancy-weighting factor for each of the remaining segments is then calculated using the Grey World method. The colour constancy adjustment factors for each pixel is finally computed by fusing the initial colour constancy of the remaining segments, regulated by the Euclidian distances of the pixel from the centroids of all remaining segments. Experimental results on both single and multiple illuminant benchmark image datasets showed that the proposed method gives a significantly higher performance to those of the state-of-the-art statistical-based techniques. Furthermore, the proposed techniques gave higher or very competitive results to those of learning-based techniques at a fraction of the computational cost.

## Figures and Tables

**Table 1:**
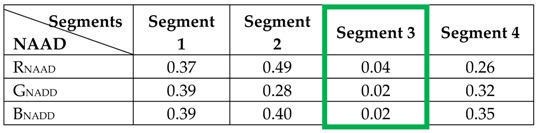
Normalised Average Absolute Difference (NAAD) values of different colour components of the image segments.

**Table 2:** Mean Opinion Score (MOS) of the proposed and the state-of-the-art techniques.

Dataset (Number of Images)	Method				
	WGE	Gisenji et al.	MIRF	Cheng et al.	Proposed CCAFIS
MLS (9 outdoor)	3.25	4.20	4.04	3.69	4.22
MIMO (78)	3.71	3.80	4.18	4.12	4.24
Grey Ball (200)	4.00	3.25	3.88	3.94	4.36
Colour Checker (100)	3.80	3.76	3.91	3.79	4.29
UPenn (57)	3.92	3.85	4.09	3.94	4.12

**Table 3:** Average mean and median recovery and reproduction angular errors of colour constancy methods’ images of the Grey Ball dataset.

Method	Recovery Error		Reproduction Error	
	Mean	Median	Mean	Median
**Statistics-based methods**				
Gray World	7.1°	7.0°	10.1°	7.5°
Max-RGB	6.8°	5.3°	9.7°	7.5°
Shades of Gray	6.1°	5.3°	6.9°	3.9°
Gray Edge-1	5.1°	4.7°	6.3°	3.6°
Gray Edge-2	6.1°	4.1°	5.8°	3.6°
Proposed CCAFIS	3.9°	4.0°	4.1°	2.6°
**Learning-based methods**				
Exemplar-based	4.4°	3.4°	4.8°	3.7°
Gray Pixel (std)	4.6°	6.2°	-	-
Deep Learning	4.8°	3.7°	-	-
Natural Image Statistics	5.2°	3.9°	5.5°	4.3°
Spectral Statistics	10.3°	8.9°	-	-
ASM	4.7°	3.8°	5.2°	2.3°

**Table 4:** Average mean and median recovery angular errors of colour constancy methods’ images of the colour checker dataset.

Method	Recovery Error		Reproduction Error	
	Mean	Median	Mean	Median
**Statistics-based methods**				
Gray World	9.8°	7.4°	7.0°	6.8°
Max-RGB	8.1°	6.0°	8.1°	6.5°
Shades of Gray	7.0°	5.3°	5.8°	4.4°
Gray Edge-1	5.2°	5.5°	6.4°	4.9°
Gray Edge-2	7.0°	5.0°	6.0°	4.8°
Proposed CCAFIS	3.9°	2.7°	4.3°	2.9°
**Learning-based methods**				
Exemplar-based	2.9°	2.3°	3.4°	2.6°
Gray Pixel (std)	3.2°	4.7°	-	-
ASM	3.8°	2.4°	4.9°	3.0°
AAC	2.9°	3.4°	-	-
HLVI BU	2.5°.	3.3°	-	-
HLVI BU & TD	2.4°	3.3°	-	-
CCDL	2.3°	3.1°	-	-
EB	2.2°	2.7°	-	-
BDP	2.1°	3.5°	-	-
CM	2.0°	2.8°	-	-
FB+GM	2.0°	3.6°	-	-
PCL	1.6°	2.5°	-	-
SF	1.6°	2.4°	-	-
CCP	1.4°	2.1°	-	-
CCC	1.2°	1.9°	-	-
AlexNet+SVR	3.0°	4.7°	-	-
CNN-Per patch	2.6°	3.6°	-	-
CNN average--pooling	2.4°	3.1°	-	-
CNN median-pooling	2.3°	3.0°	-	-
CNN fine-tuned	1.9°	2.6°	-	-
CNN + SVR	2.3°	1.4°	-	-

**Table 5:** Mean and median recovery angular error of various methods on images of the MIMO dataset.

Method	MIMO (real)		MIMO (lab)	
	Mean	Median	Mean	Median
**Statistical methods**				
Grey world	4.2°	5.2°	3.2°	2.9°
Max-RGB	5.6°	6.8°	7.8°	7.6°
Grey Edge-1	3.9°	5.3°	3.1°	2.8°
Grey Edge-2	4.7°	6.0°	3.2°	2.9°
MIRF	4.1°	3.3°	2.6°	2.6°
Grey Pixel	5.7°	3.2°	2.5°	3.1°
Proposed CCAFIS	4.2°	4.3°	2.1°	2.7°
**Learning-based methods**				
MLS + GW	4.4°	4.3°	6.4°	5.9°
MLS + WP	4.2°	3.8°	5.1°	4.2°
MIRF + GW	3.1°	2.8°	3.1°	2.8°
MIRF + WP	4.1°	3.3°	3.0°	2.8°
MIRF + IEbV	5.6°	4.3°	4.5°	3.0°

**Table 6:** Median recovery angular errors for 9 outdoor images of the multiple light source dataset.

Method	Median Error
Max-RGB	7.8°
Grey World	8.9°
Grey Edge-1	6.4°
Grey Edge-2	5.0°
Gisenji et al.	5.1°
Proposed CCAFIS	2.6°

**Table 7:** Cumulative execution time for the first 100 images of the Colour Checker benchmark image dataset for the proposed CCAFIS, statistical-based and learning-based colour constancy adjustment techniques.

Method	Time (s)
**Statistics-Based Methods**	
Gray World	2.01
Max-RGB (White Patch)	2.21
Gray Edge-1	23.67
Gray Edge-2	24.21
Proposed CCAFIS	26.37
**Learning-Nased Methods**	
Exemplar-based	2827
Gray Pixel (std)	1165
ASM	2500
